# Carbon Nanotubes Reinforced Composites for Biomedical Applications

**DOI:** 10.1155/2014/518609

**Published:** 2014-02-24

**Authors:** Wei Wang, Yuhe Zhu, Susan Liao, Jiajia Li

**Affiliations:** ^1^Department of Prosthodontics, School of Stomatology, China Medical University, Shenyang 110002, China; ^2^School of Materials Science and Engineering, Nanyang Technological University, Singapore 639798

## Abstract

This review paper reported carbon nanotubes reinforced composites for biomedical applications. Several studies have found enhancement in the mechanical properties of CNTs-based reinforced composites by the addition of CNTs. CNTs reinforced composites have been intensively investigated for many aspects of life, especially being made for biomedical applications. The review introduced fabrication of CNTs reinforced composites (CNTs reinforced metal matrix composites, CNTs reinforced polymer matrix composites, and CNTs reinforced ceramic matrix composites), their mechanical properties, cell experiments *in vitro*, and biocompatibility tests *in vivo*.

## 1. Introduction

Carbon is an important element to various sciences, from physics, chemistry, and materials science to life science, but conventional carbon formulation in the micron scale may not be the optimal implant material [1]. Then the nanomaterials such as the carbon nanotubes (CNTs), with unique electrical, mechanical, and surface properties, have captured the attention and aroused the interest of many scientists, since CNTs were discovered by Iijima in 1991 and up to now appear well suited as a biomaterial [2–7]. CNTs are substances with cylindrical structure of about 1 nm diameter and 1–10 *μ*m length, consisting of only carbon atoms. In general, CNTs contain single-wall carbon nanotubes (SWCNTs) and multiwall carbon nanotubes (MWCNTs). SWCNTs are viewed as rolled-up structures of single sheets of graphene and individual carbon structures approximately 1 nm in diameter and up to a millimeter or more in length, and MWCNTs are similar to hollow graphite fibers, except that they have a much higher degree of structural perfection, which are having a diameter of 10–200 nm [8–11]. Lu and Tsai investigated the load transfer efficiency in double-walled carbon nanotubes (DWCNTs, a hollow cylindrical structure, which contains two concentric graphene layers) using multiscale finite element modeling, and the results showed that increasing of CNTs' length can effectively improve the load transfer efficiency in the outermost layers, while the DWCNTs with incremental covalent bonds exhibit increasing load transfer efficiency in the inner layer. Besides, compared with SWCNTs, the DWCNTs still possess the less capacity of load transfer efficiency [12]. For MWCNTs, the outer graphene layers as well as the inner layers may be responsible for sustaining the applied load, and the load carrying capacity from the outermost layer to interior layers in MWCNTs associated with different interatomistic properties are waiting to be investigated thoroughly [12].

Treacy et al. measured the elastic modulus of MWCNTs to be 1TPa, on the same level of diamond. Compared with steel, the mechanical strength is 100 times higher of steel, but the density is only one sixth of the steel [13, 14]. Wang et al. studied the axial strength of MWCNTs and reported elastic modulus values ranging from 200 to 400 GPa, the bending modulus is to 14 GPa, and compression strength is about 100 GPa. The high deformation of CNTs allows it to break when tensile strength reaches 18% [15]. Iijima et al. investigated bending strength of CNTs, and their experimental results and theoretical studies have demonstrated that CNTs have extremely high tensile strengths, as high as 100 GPa [16, 17]. Depending on the outstanding quality of the CNTs, it is possible to use CNTs for composites reinforcement. It is also believed that the incorporation of CNTs into matrix materials should lead to composites with unique properties.

Compared with conventional carbon, CNTs are stronger and more flexible and have a higher tensile strength to weight ratio [18]. Since CNTs have a density even smaller than graphite due to the tube structure, and with sufficient high strength and excellent thermal and chemical stability, the CNTs material may be used as a structural material in the biomedical field [4, 19–24]. At present, CNTs are used as carriers for drug delivery and gene therapy, and CNTs have been shown effective to reinforce scaffolds for tissue engineering and regenerative medicine [25–30]. Since CNTs have pores and the pores of SWCNTs and MWCNTs were, respectively, less than 1 nm and 4–30 nm in diameter [31, 32], SWNTs and MWNTs might be available for tissue regeneration. Besides, CNTs have been used as supports for enzyme immobilization to improve biocatalyst performances such as activity, stability, and reusability [33]. CNTs can be easily separated by simple filtration [34] and enzymes can be adsorbed [35, 36] or covalently attached [37, 38] on surface of SWCNTs and MWCNTs. The study of Prlainovic showed that lipase can be successfully adsorbed on the surface of unmodified MWCNTs, and immobilized preparations were characterized, with FT-IR spectroscopy, AFM, and cyclic voltammetry [39]. As filler materials, CNTs are used to improve the properties of polymer composites [40].

To date, various composite materials have been prepared by incorporating SWCNTs or MWCNTs into a metal matrix, a ceramic matrix, or a polymer matrix (including SiC ceramic, SiN ceramic, quartz, Al_2_O_3_, and mental ceramic) [41–50]. And CNTs reinforced polymer matrix composites and CNTs reinforced ceramic matrix composites may be used as a structural material in the bone cement, bone filling material, and tissue engineering scaffolds [45–50]. Webster et al. fabricated polyurethane/CNTs composite, and the composite material possessed better electrical conductivity and mechanical properties, which can be used in neural tissue and bone [51, 52]. Deng et al. studied the use of MWCNT/chitosan (CHI) scaffolds, composed of MWCNTs (up to 89 wt%) and CHI and with a well-defined microchannel porous structure, as biocompatible and biodegradable supports for cell growth [53].

This review addresses the different synthetic methods, mechanical properties, and biocompatibility of CNTs-based reinforced composites, which may indicate that CNTs-based reinforced composites appear suited as biomaterials and may become useful scaffold materials for tissue engineering.

## 2. Fabrication of CNTs and CNTs-Based Reinforced Composites

### 2.1. Fabrication of CNTs

CNTs are generally prepared using five main synthesis methods containing ARC discharge, laser ablation, chemical vapor deposition (CVD), catalyst chemical vapor deposition (CCVD), and template-directed synthesis [54, 55]. Although arc discharge is a common method for CNTs synthesis, it is difficult to control the morphology of CNTs, such as length, diameter, and number of layers. Compared with arc-discharge and laser-ablation methods, CVD is most widely used for its low set-up cost, high production yield, and ease of scale-up [56]. CCVD is the most flexible and economic method for the production of CNTs; however, since many parameters influence the producing process, it is still very complex for precisely controlled growth of CNTs [54]. In the study of Disfani, MWCNTs produced by the catalytic carbon vapor deposition (CCVD) process were then functionalized, which were designated as CNTs-COOH, CNTs-OH, and CNTs-NH2. And different functionalized CNTs, as well as nonfunctionalized CNTs, were incorporated into a phenoxy resin via a melt mixing process [57].

### 2.2. Fabrication of CNTs-Based Reinforced Composites

#### 2.2.1. Fabrication of CNTs Reinforced Metal Matrix Composites

CNTs reinforced the strength, hardness, abrasion, and wear properties and thermal of stability of metal, and CNTs reinforced metal matrix composites are prepared through a variety of processing techniques, such as powder metallurgy, the melt casting, spray forming, electrochemical deposition, and other novel techniques. At present, CNTs as reinforcement in Fe-matrix, Cu-matrix, Mg-matrix, and Ni-matrix composite materials have been successfully fabricated [58–62]. Kuzumaki et al. produced CNTs reinforced aluminum (Al) composites by hot-press and hot-extrusion methods [63]. CNTs-Fe-Al_2_O_3_ composites have been prepared by hot pressing [64, 65].

#### 2.2.2. Fabrication of CNTs Reinforced Polymer Matrix Composites

The common fabricating methods of CNTs/polymer composites are solution mixing, melt blending, in situ polymerization, and sol-gel method [66]. Uniform dispersion of CNTs in polymer is a fundamental challenge and several factors that influence the dispersion of CNTs in a polymer matrix have to be considered in the preparation process of CNTs/polymer composites. In recent years, many polymers, such as epoxy [67–69], PMMA [70–73], PVA [74], PVC [75], PP [76], PE [77, 78], PA12 [79], and PS [80, 81], have been employed as matrices to prepare CNTs/polymer composites.

#### 2.2.3. Fabrication of CNTs Reinforced Ceramic Matrix Composites

Ceramic materials possess high temperature resistance, corrosion resistance, and better biocompatibility compared with metal and polymer. The poor mechanical properties of ceramic with regard to its brittleness and low fracture toughness restrict its use in load bearing applications [82–85]. Therefore, CNTs with excellent physical and chemical properties are added to enhance the mechanical properties of the ceramic matrix. The fabricating methods of CNTs/ceramic composites include hot pressing process (HP), hot isostatic pressing-sintering (Sinter-HIP), spark plasma sintering (SPS), microwave sintering, and high-temperature extrusion molding according to the sintering process [86, 87]. SPS method is a newly developed technique used widely since 1990 [88]. During recent years, various ceramics, composites, cermets, and other materials, including Al, Ti, and functionally graded materials (FGM), have been successfully compacted by SPS [88–96]. Compared with other sintering methods, SPS method has several advantages. The SPS method can break surface oxide layer on particles and heat them up instantly by electric spark discharges under compressive pressure. In this way, it is possible to obtain fully dense samples at relatively low sintering temperature and pressure in a very short holding time [56, 95, 97]. Besides, by rapid temperature rise, grain growth of the raw material is kept to a minimum, thus making it possible to maintain the nanotube structure in the sintered bulk CNTs. Wang et al. successfully fabricated CNTs-based composites including MWCNTs/5, 20, 25% polycarbosilane (PCS), 100% MWCNTs, and MWCNTs/40% hydroxyapatite (HA) composites by using the SPS method under different sintering conditions. In addition, Yao et al. fabricated CNTs/alumina reinforced composite by a combined process of pressureless sintering and atmosphere hot-pressing sintering [96]. Ogihara et al. synthesized the CNTs/alumina composite using pressureless sintering under vacuum and hot isostatic pressing [97].

## 3. Mechanical Properties and Biocompatibility of CNTs-Based Reinforced Composites

### 3.1. Microstructure

**Figure 1:**
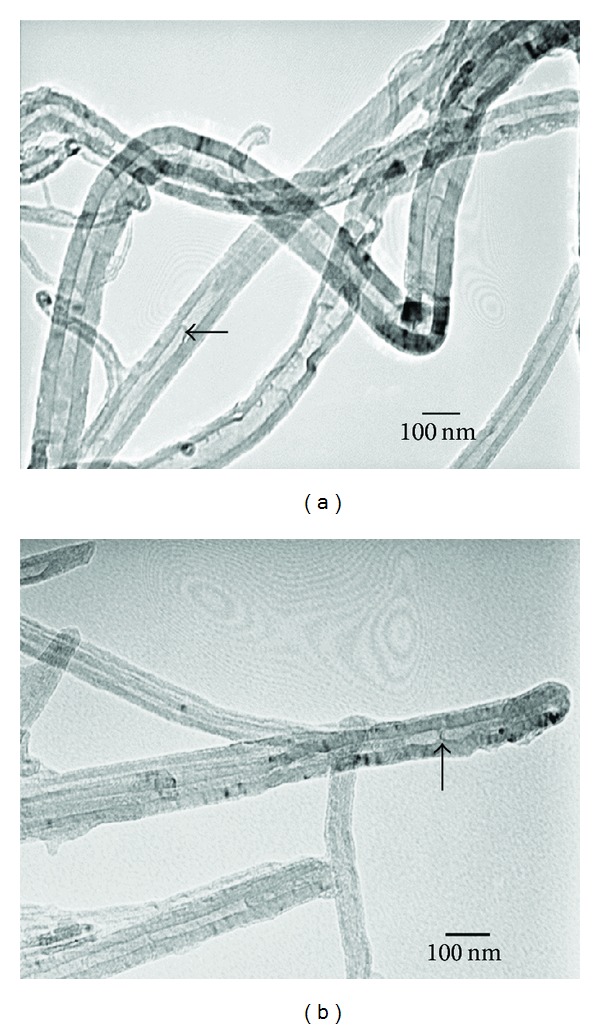
TEM images of MWCNTs starting powders and 100% MWCNTs monolith after SPS treatment [98]. (a) MWCNTs powders and (b) 100% MWCNTs monolith.

CNTs have recently gained substantial interest for their potential applications in tissue engineering due to their large ratio of surface area to volume and unique microstructure. From the TEM micrographs, MWCNTs starting powders had external and internal diameters of 20–80 nm and 10–50 nm, and the 100% MWCNTs monolith basically maintained the nanosized tube microstructure and the bamboo microstructures following SPS treatment, as indicated by the hollow arrow in Figures 1(a) and 1(b) [98].

**Figure 2:**
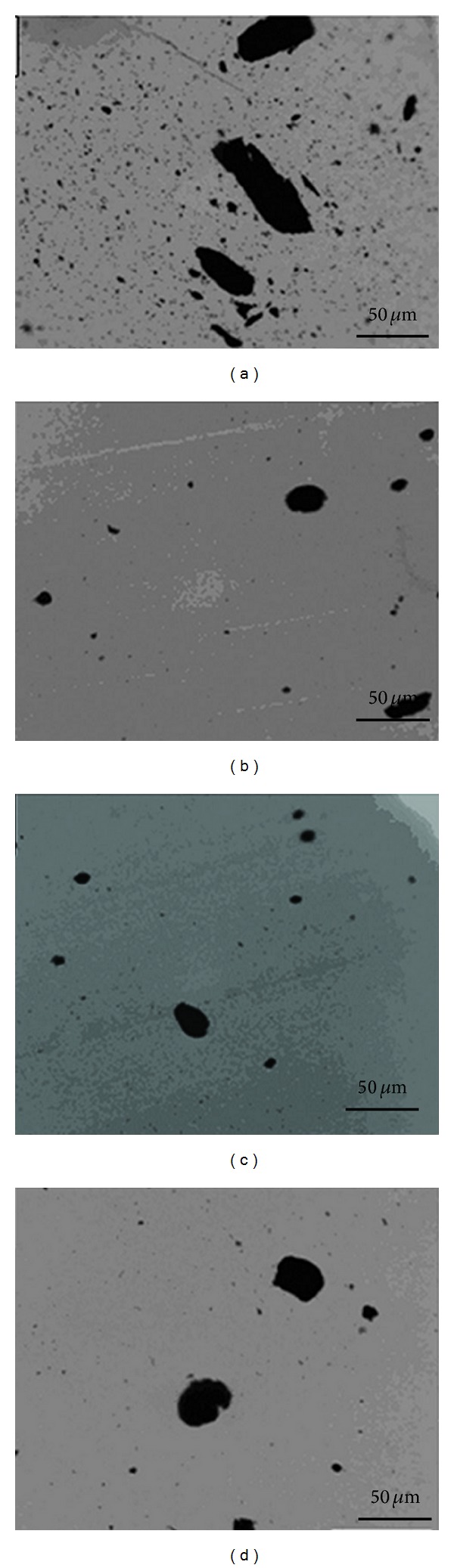
Optical microscopic images of phenoxy/MWCNTs nanocomposites containing (a) CNTs-COOH, (b) CNTs-OH, (c) CNTs-NH2, and (d) pure CNTs [57].

**Figure 3:**
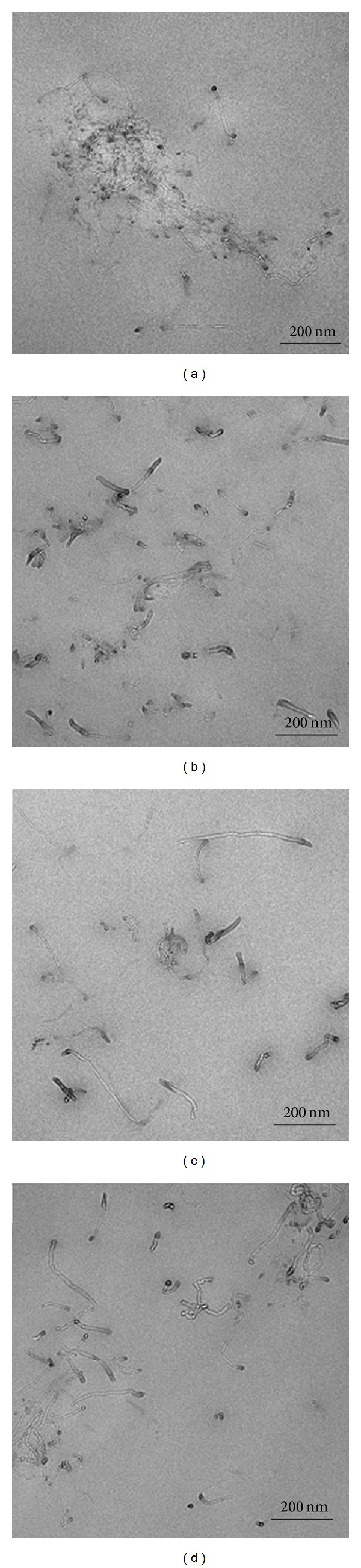
TEM images of phenoxy/MWCNTs nanocomposites containing (a) CNTs-COOH, (b) CNTs-OH, (c) CNTs-NH2, and (d) pure-CNTs [57].

For the phenoxy/MWCNTs nanocomposites, optical microscopic images were shown as in Figure 2, from which we can see the state of CNTs dispersion in phenoxy matrix for different functionalized and nonfunctionalized MWCNTs, and compared with the other composites, the agglomerates are much bigger for CNTs-COOH (Figure 2(a)) [57]. TEM images of phenoxy/MWCNTs nanocomposites were shown as in Figure 3. The size of aggregates was in the scale of 200 nm, and the size of CNTs aggregates follows the following trend: CNT-COOH>pure-CNT>CNT-OH>CNT-NH [57].

In the sintering process of MWCNTs/5, 20, and 25% PCS, nanosized SiC particles pyrolyzed from PCS during sintering worked as the binder for MWCNTs, while HA was selected as binder to consolidate MWCNTs, which has been extensively used for maxillofacial surgery, orthopedics, and implant fabrication and is one of the most compatible biomaterials owing to its similar chemical composition and crystal structure to apatite in human hard tissue such as bone and tooth [84, 85, 99]. However, the poor mechanical properties of HA with regard to its brittleness and low fracture toughness restrict its use in load bearing applications (orthopedic/dental implant) [86, 87].

### 3.2. Mechanical Properties

It has been well proved that the mechanical property of matrix could be largely enhanced by the addition of CNTs [100, 101].

#### 3.2.1. Mechanical Properties of CNTs Reinforced Metal Matrix Composites

For AZ31/CNTs composite, the maximal tensile strength and the elongation of the AZ31/CNTs composites are enhanced by 41.3% and 119.4%, respectively, and the elastic modulus and microhardness are also raised by 67.8% and 66.9%, respectively, when compared with those of the as-cast AZ31 Mg alloys [102]. Kim et al. were the first to report Cu-CNTs reinforced composites by SPS. Further rolling was performed on the composite to deform and align the CNT rich regions resulting in improved properties. SPS of Cu-CNTs nanocomposite powder, produced by molecular level mixing process, helps further improve density and mechanical properties. Enhancement in mechanical strength by 129% with addition of 5 vol% CNTs had been demonstrated [103].

#### 3.2.2. Mechanical Properties of CNTs Reinforced Polymer Matrix Composites

In previous study, carboxyl-functionalized MWCNTs were used as fillers in a polyamide 6 (PA6) matrix in order to change the effect of the material [104, 105]. Sun et al. reported that the addition of CNTs improved the storage modulus E′ and loss modulus E′′ of the PA6/CNTs composite [104]. Zomer Volpato et al. synthesized MWCNTs/PA6 composite, and incorporation of up to 2 wt% CNTs in CNTs/PA6 laminates improved the flexural stress of the laminates up to 36%, which should form hydrogen bonds between the polymer and filler or form amide bond between the free amines on the polymer and the CNTs carboxyl groups [105].

To improve the physiochemical properties of polyurethane (PU), CNTs are incorporated to add functionalities of material. For instance, Amr et al. reported that Young's modulus of CNTs/polystyrene (PS) nanocomposites was increased by 22% [106]; Jung et al. reported that the transparent PU film was incorporated with functionalized MWCNTs and found 2-fold and 10-fold increases in tensile strength and modulus, respectively, for MWCNTs/PU composite film [107]. According to the result of Tijing, the incorporation of MWCNTs increased the tensile strength and modulus of the composite nanofibers by 69% and 140%, respectively, and 62% and 78%, respectively, for composite films, and the MWCNTs/PU composites showed an improved thermal degradation behavior [108].

#### 3.2.3. Mechanical Properties of CNTs Reinforced Ceramic Matrix Composites

Yao et al. reported that the mechanical properties of the CNTs/alumina reinforced composite can be obviously improved due to the addition of the CNTs. As the increase of mass fraction of carbon nanotubes, the tensile strength and Brinell hardness of the composite are elevated and achieve the maximum of 245 MPa and 106.66 n/mm^2^, respectively, when the mass fraction of CNTs increases to 2.0 wt% [96]. Ogihara et al. synthesized the CNTs/alumina composite by direct growth of CNTs on alumina by chemical vapor deposition (CVD) and the as-grown nanocomposites were densified by SPS, and the mechanical strength was enhanced as follows: Young's modulus, 383 GPa; Vickers hardness, 19.9 GPa; Bending strength, 578 MPa [97].

For Zirconia-MWCNTs composites, the addition of MWCNTs aims to avoid the slow crack propagation and to enhance the toughness of the ceramic material used for prostheses. The sample of Zirconia MWCNTs shows higher density, lower grain size, improved toughness, and enhanced hardness, which suggested the good behavior of MWCNTs as strengthening agents for zirconia [109].

For MWCNTs/PCS composites, it is found that PCS content and sintering pressure improved the bulk density and Vickers hardness of sintered MWCNTs, and the value of mechanical properties was highest for the MWCNTs/20% PCS. The bulk density, Young's modulus, and compressive strength of the MWCNTs/20% PCS material had the highest value of 2.13 g/cm^3^, 27 GPa, and 298 MPa, which was higher than that of human bone. However, the bulk density, Young's modulus, and compressive strength of 100% MWCNTs monolith were 1.95 g/cm^3^, 20 GPa, and 249 MPa, which were very closer to those of bone (1.9 g/cm^3^, 19 GPa, and 150 MPa) and lower than those of other traditional implant materials: Ti (4.51 g/cm^3^, 120 GPa, and 500 MPa) and HA (3.15 g/cm^3^, 35 GPa, and 600 MPa) [98, 110–113]. The results showed that the 100% MWCNTs monolith could match the mechanical properties of human compact bone, which might be more suitable for implant materials than HA and Ti.

### 3.3. Biocompatibility

At present, carbon nanotubes have been extensively studied for use in biomedical applications, and biomaterials using CNTs are expected to be developed for clinical use [114–119]. Some studies showed that nanophase biomaterials had higher biocompatibility than similar micron-sized materials [5, 120]. Many studies *in vivo *and *in vitro* have investigated the biocompatibility of CNTs for biomedical applications. There are controversies on CNTs cytotoxicity, and CNTs might have adverse effects, which is ascribed to their physicochemical properties, such as structure, surface area, extent of oxidation, producing method, and concentration [121]. The toxicity of CNTs on the respiratory system is investigated. Lam et al. studied toxicity of CNTs by bronchial injection test, and the results of studies showed that 0.5 mg of CNTs can cause the death of part of mice, another part of the lungs in mice is characterized by damage granuloma [122]. In contrast, Miyawaki et al. investigated *in vitro* and *in vivo* the toxicities of carbon nanohorns (CNHs). The CNHs were found to be a nonirritant and a nondermal sensitizer through skin primary and conjunctival irritation tests and skin sensitization test. The acute peroral toxicity of CNHs was found to be quite low; the lethal dosage for rats was more than 2000 mg/kg of body weight. Intratracheal instillation tests revealed that CNHs rarely damaged rat lung tissue for a 90-day test period, although black pigmentation due to accumulated nanohorns was observed. Yet the present results suggest that CNHs have low acute toxicities [123].

Used in the scaffold, CNTs could promote cell adhesion, and MWNTs could decrease osteoclast number to inhibit bone resorption [124, 125]. When it comes to osteoblasts, CNTs did not have cytotoxicity to osteoblasts and did not have harmful effects on osteoblast differentiation or mineralization [126–128]. In addition, nonfunctionalized SWCNTs had little toxicity to cell such as decreasing the viability and number of cells [129]. It is reported that there was no acute toxicity or adverse reaction for functionalized CNTs; however, the severe tissue deposition and inflammatory response were observed for pristine CNTs. Tang et al. modified the CNTs with macromolecules (polyethylene glycol PEG), and the results indicated that the synthesized CNTs are very biocompatible, exhibiting no differences from normal control groups, and in other words, shorter pristine and polymer functionalized MWCNTs have a significant potential for biomedical applications as efficient carriers for diagnostic, therapeutic, or cell-specific targeting molecules [130]. Ahn et al. investigated the incorporation of MWCNTs into calcium phosphate cements (CPC) and evaluated the bioactive nature of CPC-MWCNTs hybrid the osteogenic differentiation capacity as bone grafting materials, using proliferation and differentiation of MC3T3-E1 cells, the result of which showed that CPC-MWCNTs hybrid which promoted the osteogenic differentiation of osteoblasts could serve well as bone repairing graft material [131]. Zomer Volpato et al. synthesized PA6/MWCNT and investigated the effect of the addition of CNTs on the cell-material interactions and found that the proliferation and activation of MG63 cell line osteoblasts were enhanced due to surface modification caused by the filler addition compared to the purely PA6 networks [105]. The result of Ogihara et al. about cell attachment of CNTs/alumina composite indicated that CNTs/alumina composite had more favorable cell attachment properties, and CNTs at the surface of the implant did not inhibit attachment [97].

**Figure 4:**
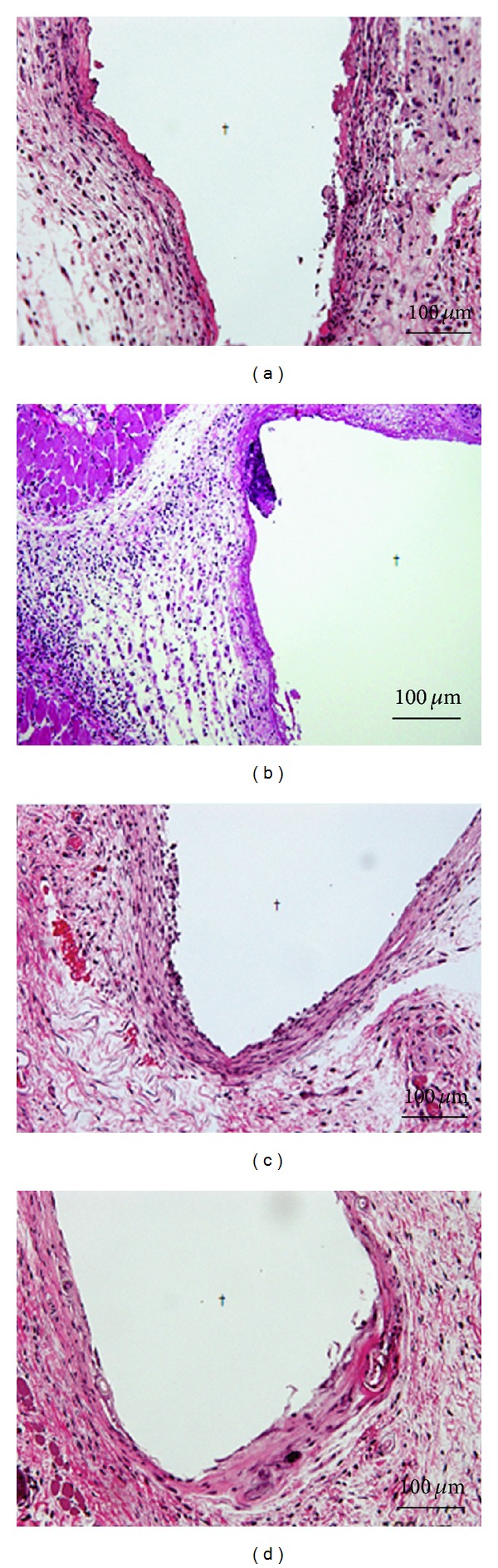
Tissue images around alumina ceramic and CNTs/alumina composites embedded in the subcutaneous tissue of mice: (a) alumina ceramic after 1 week, (b) CNTs/alumina after 1 week, (c) alumina ceramic after 4 weeks, and (d) CNTs/alumina after 4 weeks [97].

Meanwhile the subcutaneous tissue reactions and bone tissue reactions were evaluated for the alumina ceramic and CNTs/alumina composite, and found that inflammatory cells were observed around the composites after 1 week, however, severe inflammatory reactions were not observed (Figures 4(a) and 4(b)) [97]. And after 4 weeks, thin fibrous capsules attached to alumina ceramic had been formed, and the inflammatory reaction had disappeared. Similar phenomenon was observed on the CNTs/alumina composite (Figures 4(c) and 4(d)) [97].

Yokoyama et al. investigated the biological behavior of hat-stacked carbon nanofibers (H-CNFs) in the subcutaneous tissue of rats, and the results showed that H-CNFs were englobed by fibrous connective tissue with little inflammation [27]. But Muller et al. found that CNTs have the potential to cause serious inflammatory and fibrotic reactions by studying rats exposed to respirable CNTs particles [132]. Colvin reported that the pulmonary toxicity of CNTs was not obvious as granulomas which were not commonly observed in rat lungs instilled with CNTs [133]. Additionally, the study of Kumar et al. has revealed that the chemical state of the surface of CNTs may strongly influence tissue response [134]. The influence of catalytic particles, like Fe and Ni, applied during the synthesis of CNTs on the toxicity of CNTs has been reported [30].

**Figure 5:**
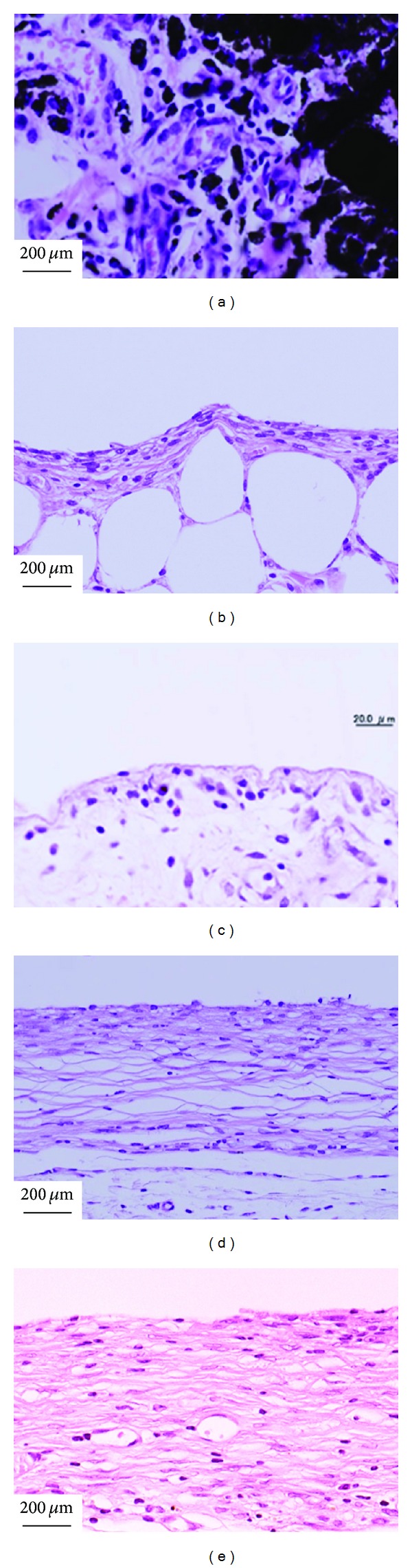
Tissue responses at one week after implantation [98, 110–112]. (a) MWCNTs powders, (b) 100% MWCNTs monolith, (c) MWCNTs/5% PCS, (d) MWCNTs/20% PCS, and (e) MWCNTs/25% PCS.

**Figure 6:**
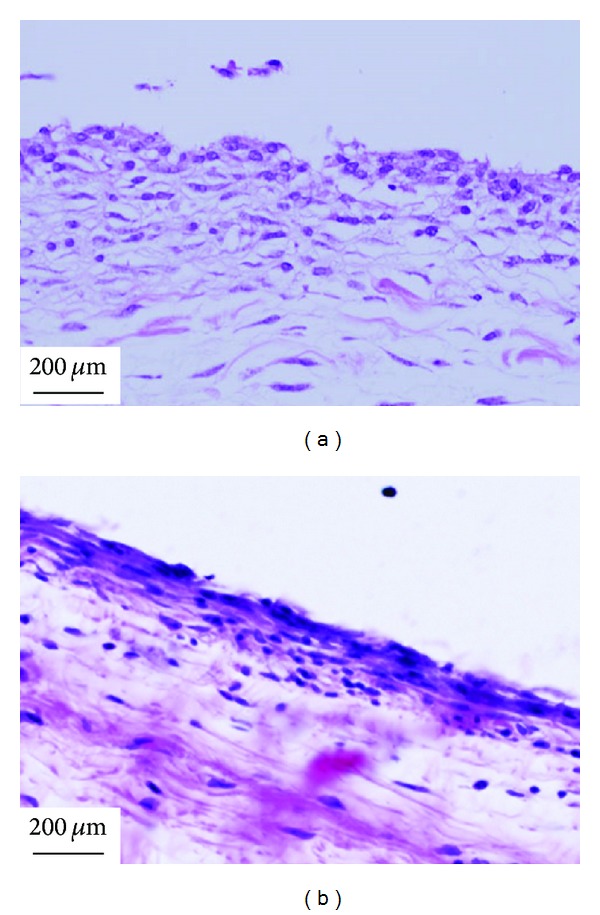
Tissue responses at 4 weeks after implantation [98, 111]. (a) MWCNTs/20%PCS and (b) 100% MWCNTs monolith.

The inflammation of MWCNTs powders is most serious in the soft tissue, which may be due to that the dispersed powder easily caused body response. At 1 week after the implantation in the soft tissue of rats, MWCNTs powders were surrounded by granulation tissue with many macrophages and foreign body giant cells (Figure 5(a)) [110], which was consistent with the study of Warheit et al., who have demonstrated that pulmonary exposures to CNTs in rats produced multifocal granulomas that consisted of macrophage-like multinucleate, [135]. However, no severe inflammatory response was observed around MWCNTs/PCS composites with different percentage of PCS and 100% MWCNTs monolith. For the response in subcutaneous tissue, there was a difference dependent on the content of PCS in the early implant stage; the degree of inflammation was influenced by SiC pyrolyzed from PCS. At 1 week after surgery, inflammatory response around MWCNTs/5% PCS (Figure 5(c)) was milder than that around MWCNTs/25% PCS (Figure 5(e)) [110]. MWCNTs/20% PCS was covered by relatively thick fibrous connective tissue including many cells with large cytoplasm like fibroblasts, fibroblasts with spindle-shaped cytoplasm, and some inflammatory round cells (Figure 5(d)) [111], and an inflammatory reaction around the 100% MWCNTs monolith was observed at 1 week after implantation in subcutaneous tissue (Figure 5(b)) [98]. But at 4 weeks after implantation, the MWCNTs/20% PCS and 100% MWCNTs monolith were covered by loose fibrous connective tissue, and inflammation around materials was slight in comparison to that at 1 week (Figures 6(a) and 6(b)) [98, 111]. The inflammatory reaction after one-week implantation is normal for the short period that immediately follows an implantation treatment.

**Figure 7:**
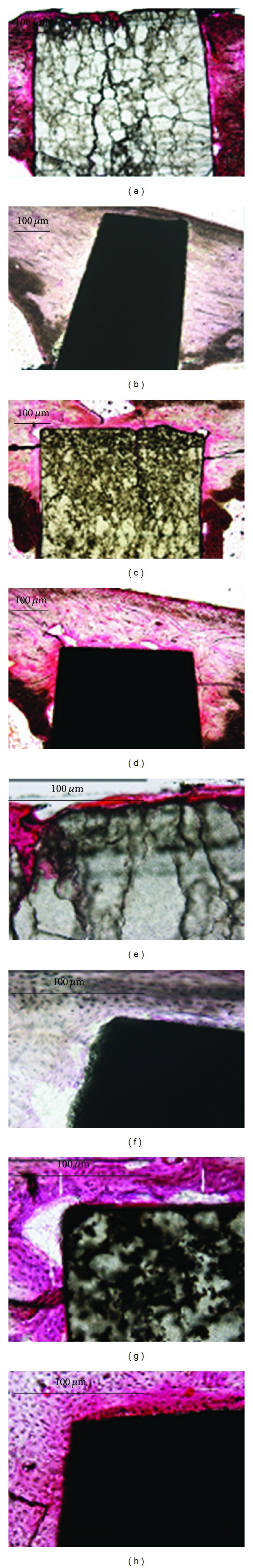
Enlarged image of the border between the specimen and the bone (200x). (a and e) Alumina ceramic was implanted after 12 weeks (40x, 220x); (b and f) CNTs/alumina composite was implanted after 12 weeks (40x, 220x); (c and g) alumina ceramic was implanted after 24 weeks (40x, 220x); (d and h) CNTs/alumina composite was implanted after 24 weeks (40x, 220x) [97].

The images of bone tissue reactions after alumina ceramic or CNTs/alumina composite implanted in rabbit femurs were shown as Figure 7 [97]. At 12 weeks, new bone was found around the composites and the fibrous capsule between the composites and the bone was rarely observed (Figures 7(a), 7(b), 7(e), and 7(f)). At 24 weeks, the entire circumference of the specimen had attached to the bone tissue without gaps, and composites were completely incorporated into the bone and the bone defect was repaired (Figures 7(c), 7(d), 7(g), and 7(h)). These results showed that the bone tissue compatibility of CNT/alumina composite is comparable with that of alumina ceramic.

**Figure 8:**
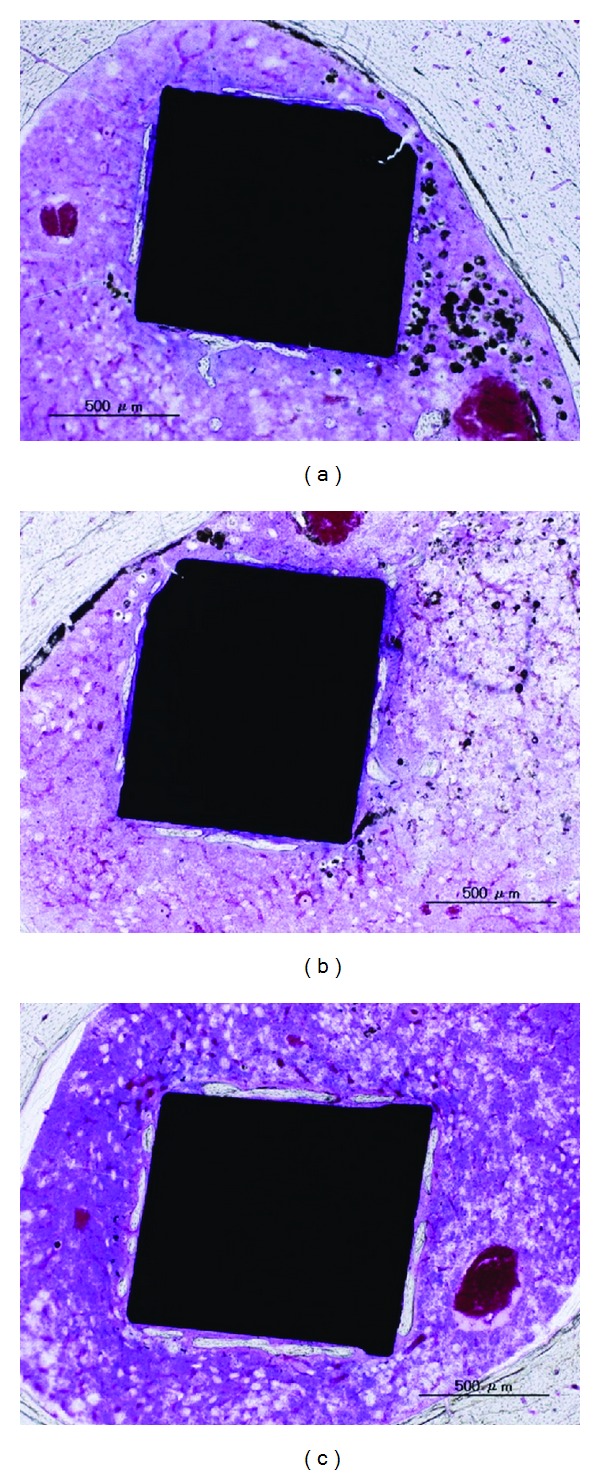
Osteogenesis of (a) MWCNTs/20% PCS, (b) 100% MWCNTs monolith, and (c) MWCNTs/40%HA in the femur at 4 weeks [98, 111, 112].

For the response in bone tissue, after implantation for 4 weeks in the femur, part of the newly formed bone attached to MWCNTs/20% PCS directly (Figure 8(a)), lamellar newly formed bone was observed around the 100% MWCNTs implant (Figure 8(b)), and a large of newly formed bone was observed around the MWCNTs/40% HA composites as shown in Figure 8(c), and the newly formed bone was attached to the implant directly [98, 111, 112]. The MWCNTs/PCS composite had very little prophlogistic effect and possessed osteoconductivity. Similar *in vitro* results were described by Elias et al. who reported that carbon fiber compacts improved the growth of osteoblasts compared to conventional carbon fiber [120]. However, the osteoconductivity was influenced by the PCS content, and the amount of the newly formed bone was least in MWCNTs/20% PCS and most in MWCNTs/40% HA. HA was added for improving the biocompatibility of MWCNTs materials. HA is widely accepted coating for orthopedic implants since 1980 due to its excellent biocompatibility and bioactivity properties [133, 136]. And many composites containing HA were fabricated and show good biocompatibility [137, 138]. MWCNTs/HA composites possessed better osseointegration than pure MWCNTs as we expected.

## 4. Conclusions and Perspectives

Nanoscale substances like CNTs could be potential applied in almost all the walks of life: media, entertainment, communication, transport, health, and environment, especially in the nanobiomedical field [53]. CNTs, with a range of unique properties, appear suited as biomaterials and may become useful scaffold materials for tissue engineering. Reinforcing scaffolds with CNTs has been suggested to be an effective means of developing engineering materials for tissue regeneration. These reinforced scaffolds have been largely applied for not only hard tissue but also soft tissue repair. However, their safety and effectiveness as biomaterials are still unclear. More and more interests were emerged in CNT-based composites, including the synthesis of the composites and their mechanical properties, cell experiments *in vitro*, and biocompatibility *in vivo*. From previous studies, we could find that there were many methods for composing the variable CNTs-based composites under different synthetic conditions. Those composites with adjustable mechanical properties could be used for different usages, such as tissue engineering, delivery of genes and drugs, scaffold, implant, or as filler in other composites to improve their mechanical properties. Besides, we found that the mechanical property of 100% MWCNTs monolith was most close to that of human bone. Moreover, in the animal experiments, no severe inflammatory response such as necrosis and no toxicity for soft tissue and bone regeneration were observed around most CNTs-based composites. The weak inflammatory reaction in short term after implantation was normal for the short period that immediately followed an implantation treatment, and the inflammation could be reduced with the extension of experiment time. The MWCNTs/40% HA composites possessed better osseointegration than other composites.

Although modified CNTs might not represent certain original structure and properties of CNTs, it is still possible for the modified CNTs-based composites to further improve their biocompatibility and effectively reinforce their mechanical properties. Above all, although there is still a lot of works to do, the CNTs-based reinforced composites will be not only applicable as artificial bone implant materials, but also for other biomedical applications potentially rewards opportunities to develop the next generation of engineered biomaterials in the future, such as tissue engineering, cell therapy, drug delivery, and diagnostic device.
